# High-Intensity Focused Ultrasound Treatment for Advanced Pancreatic Cancer

**DOI:** 10.1155/2014/205325

**Published:** 2014-06-26

**Authors:** Yufeng Zhou

**Affiliations:** School of Mechanical and Aerospace Engineering, Nanyang Technological University, 50 Nanyang Avenue, Singapore 639798

## Abstract

Pancreatic cancer is under high mortality but has few effective treatment modalities. High-intensity focused ultrasound (HIFU) is becoming an emerging approach of noninvasively ablating solid tumor in clinics. A variety of solid tumors have been tried on thousands of patients in the last fifteen years with great success. The principle, mechanism, and clinical outcome of HIFU were introduced first. All 3022 clinical cases of HIFU treatment for the advanced pancreatic cancer alone or in combination with chemotherapy or radiotherapy in 241 published papers were reviewed and summarized for its efficacy, pain relief, clinical benefit rate, survival, Karnofsky performance scale (KPS) score, changes in tumor size, occurrence of echogenicity, serum level, diagnostic assessment of outcome, and associated complications. Immune response induced by HIFU ablation may become an effective way of cancer treatment. Comments for a better outcome and current challenges of HIFU technology are also covered.

## 1. Pancreatic Cancer

Pancreas is an essential gland organ in the digestive and endocrine system, producing hormones (i.e., insulin, glucagon, and somatostatin) into the bloodstream and secreting pancreatic juice to the small intestine or gut. Although pancreatic cancer is the twelfth most common cancer for humans, its mortality ratio is as large as 98% [1] and is the fourth leading cause of cancer death. 338,000 new cases were diagnosed in 2012, and the estimated 5-year prevalence of pancreatic cancer is 4.1 per 100,000 in the world. About 55% of pancreatic cancer cases occurred in more developed countries, such as Northern America and Europe, while Africa and Asia have the lowest incidence. The American Cancer Society estimates that about 46,420 people (23,530 men and 22,890 women) will be diagnosed with pancreatic cancer and among them 39,590 people (20,170 men and 19,420 women) will die in the United States in 2014. In Europe, the corresponding death number is estimated to be 80,266 people (40,069 men and 40,197 women) [2]. A comprehensive genetic analysis of 24 pancreatic cancers showed an average of 63 genetic alterations. These alterations defined 12 core cellular signaling pathways and processes in 67 to 100% of the tumors, which suggests the complexity of pancreatic tumor's genetics [3].

Owing to the absence of specific symptoms and effective screening, most of pancreatic cancers are diagnosed at the late stage (TNM III or IV) with locally advanced (60%) and metastatic disease (20%). Only about 15 to 20% of patients can undergo curative surgical resection, and the 5-year survival is just 30%. Surgery also associates a considerable risk of morbidity and mortality. If the tumors are involved with superior mesenteric artery and/or celiac axis even in the early stages, they are also generally considered unresectable. Liver, peritoneum lungs, bones, and brain are the popular sites of metastases in pancreatic cancer sorted by their feasibilities. Metastases to muscle, skin, heart, pleura, stomach, umbilicus, kidney, appendix, spermatic cord, and prostate are occasionally observed. Gemcitabine is the gold standard drug for advanced pancreatic cancer; however, its clinical benefit response (CBR) is 12 to 23.8%, and the median survival is only prolonged by a further 10 days. Erlotinib is the only targeted drug approved by the Food and Drug Administration (FDA). Chemotherapy, radiotherapy, and targeted drug are rather ineffective for this malignancy [4]. The median survival of pancreatic cancer patients is less than 3 months without therapy and less than 6 to 12 months with therapy. Overall 1-, 3-, and 5-year survival of pancreatic cancer patients are 16%, 5%, and 4%, respectively [5]. Therefore, alternative solution for inoperable cases is strongly desired.

Most pancreatic cancer patients have severe abdominal pain and significantly decreased quality of life, which is mainly owing to the proximity of the pancreas to the duodenum, liver, stomach, jejunum, and transverse colon. The pain is usually dull and radiates to the waist, sometimes sharp and severe and could be both neuropathic and inflammatory because of both tumor expansion and invasion of the celiac and mesenteric plexus. Sleep and appetite will be affected when an advanced tumor invades the solar plexus. Pain relief for advanced pancreatic cancer patients to enhance their quality of life is an ongoing challenge. Although an increasing number of effective opioids are available for the pain mitigation, these analgesics have obvious adverse effects, such as vomiting, constipation, and dysphoria to respiratory depression. Chemotherapy and radiotherapy are not very effective in pain relief, and the associated side effects are very serious.

Although radiofrequency ablation (RFA), percutaneous ethanol injection, cryoablation, microwave ablation, and laser-induced interstitial thermotherapy have been used widely to induce coagulative necrosis for various solid tumors, it remains difficult to use them to manage those in difficult locations, such as pancreatic malignancies. Precise ablation in advanced pancreatic cancer is necessary owing to the high propensity of complications in the surrounding pancreatic parenchyma; otherwise, a pancreaticocutaneous fistula and severe pancreatitis will be produced. So, none of them is standardized for pancreatic malignancies. RFA was used for coagulation of unresectable pancreatic cancer, but two patients died from severe complications in 20 treated cases [6].

## 2. HIFU Technology and Mechanism

Interaction of ultrasound at great intensity with tissue and, subsequently, physical and biological changes has been investigated for decades [8]. Therapeutic ultrasound can be classified based on the intensity; the low intensity (0.125–3 W/cm^2^) is to stimulate physiological responses or to accelerate the transport of drugs across the skin while the high intensities (>5 W/cm^2^) intend to selectively destroy tissue in a controlled fashion. By focusing high power ultrasound beams inside the human body away from the source, almost complete necrosis of tumor lying within the focal region, especially those in difficult locations, could be achieved successfully without damage to the intervening tissue. Ultrasound surgery was first proposed as a destroying tool for neurosurgical research [9]. In the 1950s, Fry brothers applied high-intensity focused ultrasound (HIFU) to treat 50 Parkinson's disease patients [10], and the first case of breast cancer by HIFU was reported in 1961 [11]. Ultrasound hyperthermia was utilized for the treatment of glaucoma in the 1980s [12]. The advent of clinical imaging and computer control techniques in the early 1990s made practical implementation of HIFU feasible and acceptable. In 1996, 20 cases of ablation of superficial bladder cancer using HIFU were reported [13]. Wide application of HIFU in clinics began from successful treatment on a patient with osteosarcoma in Chongqing, China, in 1997. Over the past 15 years, more than 30,000 cases of uterine fibroids and cancers in the liver, breast, pancreas, bone, and kidney have been performed using HIFU with promising results [14].

The major advantages of HIFU technology are summarized as follows, but not limited [15, 16]. HIFU is a completely noninvasive procedure. There is no requirement of incisions or transfusions in the tumor ablation; thus the risks and complications associated with invasive procedures could be minimized. Acoustic intensity is only at a high level in the focal region, but not in the intervening tissue, significantly reducing the side effects, such as skin burns, discomfort, and collateral damage (i.e., hemorrhage). A broad range of tumors could be treated if the acoustic transmission window is available. Because of no ionizing radiation involved with HIFU, theoretically, there is no limitation on the number of sessions. HIFU treatment can be performed with the patient either fully conscious, lightly sedated, or under light general anesthesia. Most importantly, HIFU offers an alternative for patients who do not have any other option available.

HIFU ablation is performed under the guidance of either magnetic resonance (MR) or ultrasound (US) imaging. Magnetically compatible HIFU transducers have been developed, and MR guidance (MRgHIFU or MRgFUS) allows cancer targeting, assessment of tissue damage, and treatment monitoring by thermometry. MRI is sometimes superior in obese patients (limited to <113 kg for the gantry), but more expensive and labor-intensive. The MR thermometry has the typical temporal frame rate of 1 to 4 seconds and spatial resolution of 2 mm × 2 mm × 6 mm. Therefore, it may be more suitable for slow heating. MRgHIFU has already been approved by the FDA for clinical therapy of uterine fibroids and breast cancer. In comparison, ultrasound-guided treatments (USgHIFU) can check the acoustic conditions in the HIFU propagation path using the same energy modality and examine the changes of echogenicity in the B-mode image in real time but cannot display the temperature maps. Elastography in sonography and MRI can measure the tissue stiffness and have the potential of assessing the lesion formation.

Pathological examination illustrates clear evidence of homogeneous coagulative necrosis, cellular destruction, pyknotic nuclei or nuclei shrink, and cell debris in the target region. The boundary between the lesion and surrounding tissue is extremely sharp, comprising only a few cell layers (~50 *μ*m). Granulation tissue, immature fibroblasts, inflammatory cells, and new capillaries are found in the margin. Small vessels (<2 mm in diameter) in the tumor, including branches of arteries and veins, are heavily destructed, which is conformed by the disappearance of endothelial cell nuclei, indistinction of cellular margins, and disruption of junctions between individual cells. Scattered intravascular thrombi are often found in the destructed vessels. As a result, there is reduced or no blood circulation in the HIFU ablated tumor and a thin peripheral rim of contrast enhancement around the coagulative necrosis. Thermolysis in the capillary is not as effective as in larger vessels [17].

Mechanisms of HIFU are a synergy of thermal effects, mechanical effects, and biological effects [14]. The quick temperature rise over 70°C within seconds in the focal region causes necrosis, liquefaction, and fibrosis of tissues. Although the majority of the initial cell death is due to necrosis from thermal injury, HIFU can also induce apoptosis, which is the major mechanism of cell death in hyperthermia and occurs at lower thermal dose than thermal necrosis. In apoptotic cells, the cell nucleus destructs with rapid DNA degradation by endonucleases by itself [18]. Tissue can be regarded as viscous fluid contained by membranes. When an acoustic wave propagates through it, relative displacement of tissue layers and directional motion or microstreaming of the fluid will occur. High shear forces produced by the microstreaming can cause transient damage to cell membranes. Viscous friction of different layers of fluid then results in the temperature elevation. bubble cavitation, the dynamics of a gas cavity with response to the alternating compressive and rarefractional acoustic pressure, is a common phenomenon in the ultrasound therapy. Gas cavity works as an effective enhancer for heat deposition, but higher concentration will lead to the change of lesion from a cigar shape to a tadpole with the head moving towards the source, which makes the control of ablation difficult and unpredictable despite enhanced therapy efficiency. If the temperature is close to 100°C, boiling in tissue may occur. A vaporized cavity with complete tissue lysis may be produced by the motion of the large boiling bubble but without protein denaturation [23].

HIFU ablation for patients with early stage cancer is curative, and a normal tissue margin is set to be about 1.5 to 2.0 cm. In contrast, it is palliative for those with advanced cancer, impeding tumor growth and improving the quality of life. After HIFU ablation, 5 to 10% of patients are under a mild fever (~38.5°C) for about a week. Several patients with huge hepatocellular carcinoma had severe fever as high as 39.5°C for 2 to 3 weeks. The severity and duration of fever seem to be directly related to the ablated tissue volume. 20 to 30% of patients experience slight and mild local pain within 1 week after HIFU ablation, but only 5 to 10% of them are given oral analgesics for 3 to 5 days. Nerve fiber was also damaged by HIFU on 4 cases of malignant bone tumor. However, nerve functions (e.g., sensation and motion) recovered completely in two patients and partially recovered within 1 year in the others. HIFU can relieve the tumor-origin pain, which is not well controlled by antineoplasty and pharmacology successfully in patients [24]. Altogether, HIFU can destroy tissue, kill tumor cells, restrain the malignant proliferation, reduce pain, and prevent metastasis.

## 3. HIFU Application on Pancreatic Cancer

**Figure 1:**
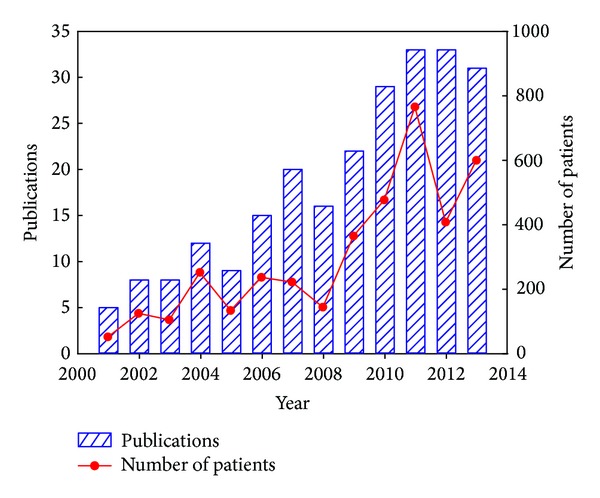
The number of publications and advanced pancreatic cancer patients treated with HIFU or in conjunction with chemotherapy or radiotherapy from 2001 to 2013.

HIFU has been used as a palliative approach for advanced pancreatic cancer (TNM stages II–IV) mostly in China since the late 1990s; Korea and Japan also adopted this modality; a few cases in Europe were reported but none in the USA because of no approval by the FDA now. Clinical cases were reported since 2001. There are in total 241 papers on HIFU application of advanced pancreatic cancer in clinics and review (mostly in Chinese) till 2013 as shown in Figure 1. The total number of patients treated by HIFU alone, HIFU with chemotherapy, and HIFU with radiotherapy is 3022 (77.74%), 668 (17.19%), and 197 (5.07%), respectively. Inclusion criteria usually are evidence of pancreatic cancer confirmed pathologically with either biopsy in initial laparotomy or sonography guided fine-needle biopsy or diagnosed by computed tomography (CT) or positron emission tomography (PET)/CT and serum analysis; presence of inoperable pancreatic cancer on the basis of surgical consultation or refusal to undergo pancreaticoduodenectomy or other treatments; minimum diameter of a solid tumor (≥1.0 cm); Karnofsky performance scale (KPS) score of at least 70%; adequate bone marrow (white blood cell count 42500/mL, platelet count 480,000/mL, and haemoglobin 48 g/mL), renal (serum creatinine concentration <1.5 mg/dL, blood urea nitrogen <20 mg%), and hepatic functions (serum transaminase level <2× the upper normal range) except hyperbilirubinemia due to obstructive jaundice; no palliative antitumor treatments have been performed in the previous 3 months. Exclusion criteria are the intolerance to HIFU treatment; radiotherapy or chemotherapy administered in the last 3 months; life expectancy <3 months; the tumor invading the duodenal wall; unstable hematogenic parameters; severe and active infection; and the patient having jaundice owing to biliary obstruction. Men patients are about 1.7 folds more than women; patient's age ranges from 15  to 89 years with a mean value of 60.8  years; cancers in the pancreas head are a little more than those in the body and tail; most patients have TNM-III and IV cancers; cancer size ranges from 2 cm to 11.9 cm with a mean value of 4.76 cm as listed in Table 1. It is important to note that detailed information about patients and cancer is not released in every clinical report.

Vital signals, such as respiration and heart rate, blood pressure, and oxygen and carbon dioxide saturation, should be monitored during the HIFU ablation. Anesthesia may be used either to avoid the painful experience or to guarantee immobilization of the target [19]. HIFU is usually carried out as a day case procedure, and average of 6.7 sessions are carried out on patients. Substantial reduction of tumor-related pain can be achieved in most cases even after one HIFU session. Pain relief, including complete relief (CR) and partial relief (PR), is about 71.33% in reported 1938 cases as listed in Table 2. The quality of life, such as appetite, sleeping, and mental status, is improved in most cases and the mean clinical benefit rate (CBR) in 508 cases is 71.06%. The mechanism of pain relief is not fully understood but hypothesized to the following mechanisms: the nerve fibers in the tumor are damaged or undergo apoptosis by the thermal effects; the targeted solar plexus may be inactivated to block the pain signal to be transferred to the brain; the pressure on the nerve applied by the tumor would be reduced due to tumor shrinkage. However, HIFU has less or no effect on the relief of obstructive pain. Average KPS increase by HIFU in reported 290 cases is about 1.5 folds. Survival is evaluated by means of the Kaplan-Meir method. In 806 cases, the median survival is 10.03 months. HIFU can kill tumor cells and block blood supply. Subsequently, potential micrometastases and lymph metastasis can be reduced, but not completely removed.

The presence of scattered intravascular thrombi after HIFU ablation will lead to malabsorption of tissue necrosis and slow atrophy of the cancer fibrosis. Structures around the pancreas determine that HIFU ablation on the pancreatic cancer is mostly palliative in nature and will not conformally reduce it as for the other solid tumors. As a result, the size of ablated tumors may not be significantly reduced but may even be increased in the short term due to the edema on the edge, which is shown in Table 3. Therefore, the pancreatic cancer size cannot be used to evaluate the efficacy of HIFU. In addition, the feasibility of echo in the target varies significantly in the clinical reports, from 0% to 100%. Enhanced echogenicity is mainly due to the presence of cavitation or boiling bubbles, and its size is smaller than the actual size of the thermal lesion.

**Figure 2:**
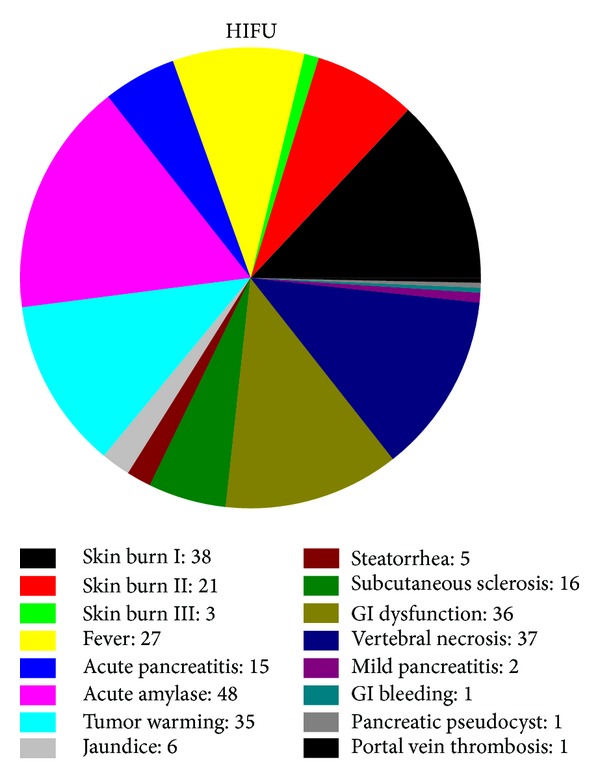
Summary of the complications found in HIFU ablation for advanced pancreatic cancer.

**Figure 3:**
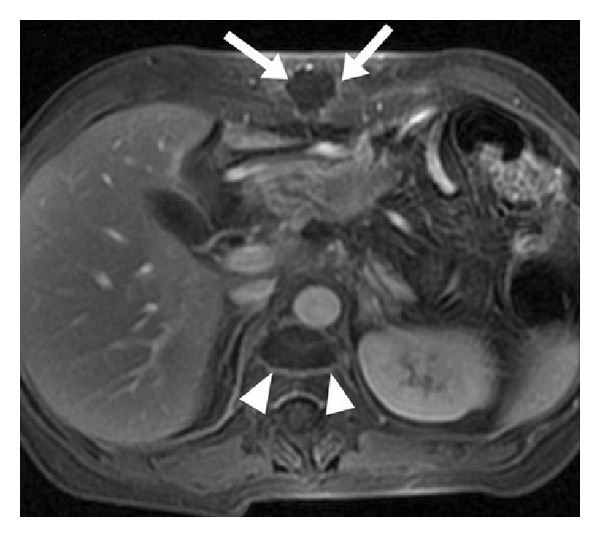
Large rim-enhancing areas of fat necrosis (arrow head) and vertebral body necrosis (arrow) along the ultrasound propagation path in a pancreatic cancer patient two weeks after HIFU ablation in fat-saturated T1-weighted magnetic resonant image after gadolinium infusion (used with permission [7]).

**Figure 4:**
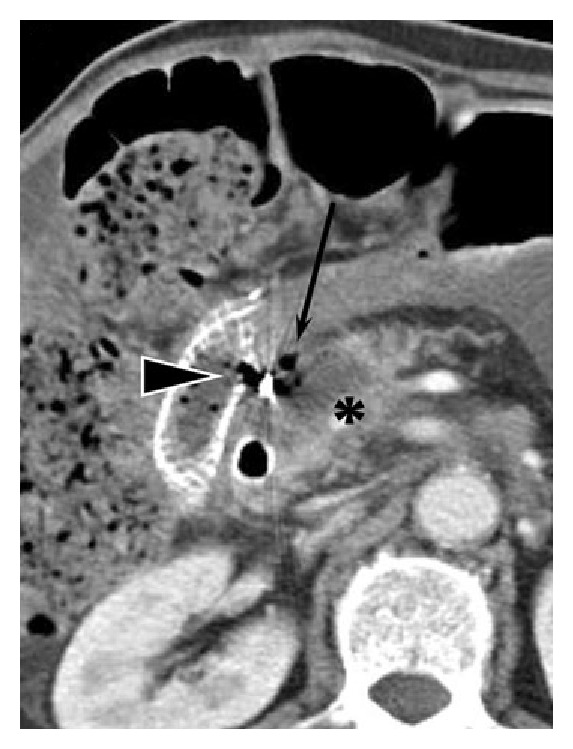
Necrosis (asterisk) in the pancreas head with rim enhancement, a fistula between the pancreatic tumor and the adjacent bowel with the mottled air densities (long thin arrows), and communication between the duodenum and the ablated cavity via focal disruption of the duodenal stent (arrowhead) in a follow-up CT after HIFU ablation (used with permission [7]).

Vital signs, liver and kidney function, skin burns, local reactions, and systemic effects are monitored and recorded before, during, and after HIFU. All of the side effects associated with HIFU ablation and reported in the published papers are summarized and shown in Figure 2. Most of them are moderate and minor complications, such as first and second degree superficial skin burns, edema, fever, tumor warming, gastrointestinal (GI) dysfunction (e.g., abdominal distension and anorexia with slight nausea), and mild abdominal pain in the treated regions [25]. The subcutaneous layer and skin are occasionally thickened and swollen, and subsequently the echogenicity is increased. Those minor complications are inevitable and mostly associated with ultrasound itself. Pancreatitis is a critical concern because HIFU can mechanically lyse cells and release pancreatic enzymes. However, pancreatic cells do not undergo lysis in thermal fixation until the intracellular enzymes have been completely denatured and inactivated, which minimizes the risk of pancreatitis in HIFU ablation. 15 cases of pancreatitis were acute and recovered usually within a week. In a study of 35 pancreatic cancer patients, all of them had vertebral body necrosis of the anterior half and 10 patients had subcutaneous fat necrosis as identified by MRI (see Figure 3) [7]. All cases were asymptomatic with no need for further treatment. Patients with extrahepatic biliary obstruction were inserted with intestinal metal stent before HIFU treatment to reduce jaundice; no deformation, displacement, or occlusion happened to the stent after HIFU treatment. One patient had portal vein thrombosis and was hospitalized for 7 days [26]. Further compression on the portal vein by the edematous tumor after HIFU ablation may be the reason of inappropriate blood clotting. A large pseudocyst surrounded by inflammatory changes was found in the mesentery anterior to the pancreas, which may be caused by the delayed perforation of the cyst near the pancreatic tumor due to damage of the cystic wall [25]. Third degree skin burns were found in the early application of HIFU and could be avoided after appropriate use of water balloon and careful examination of the coupling condition [24]. Two cases of mild pancreatitis were also found in the preliminary application [27]. One transient upper GI bleeding was observed due to a nasogastric tube. Two patients had tumor-duodenal fistulas with severe abdominal pain (see Figure 4) [28]. However, most major complications could be avoided through careful treatment planning and monitoring during the procedure. A low energy is preferred if the necrosis production is effective.

**Figure 5:**
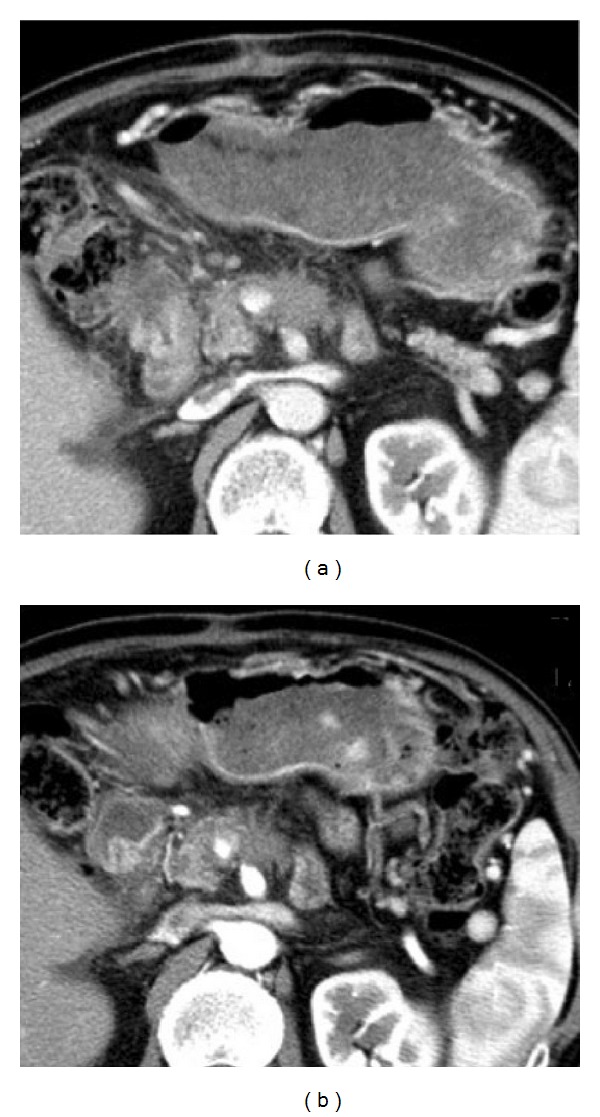
CT imaging shows no apparent change of pancreas (a) before and (b) after HIFU therapy (used with permission [18]).

**Figure 6:**
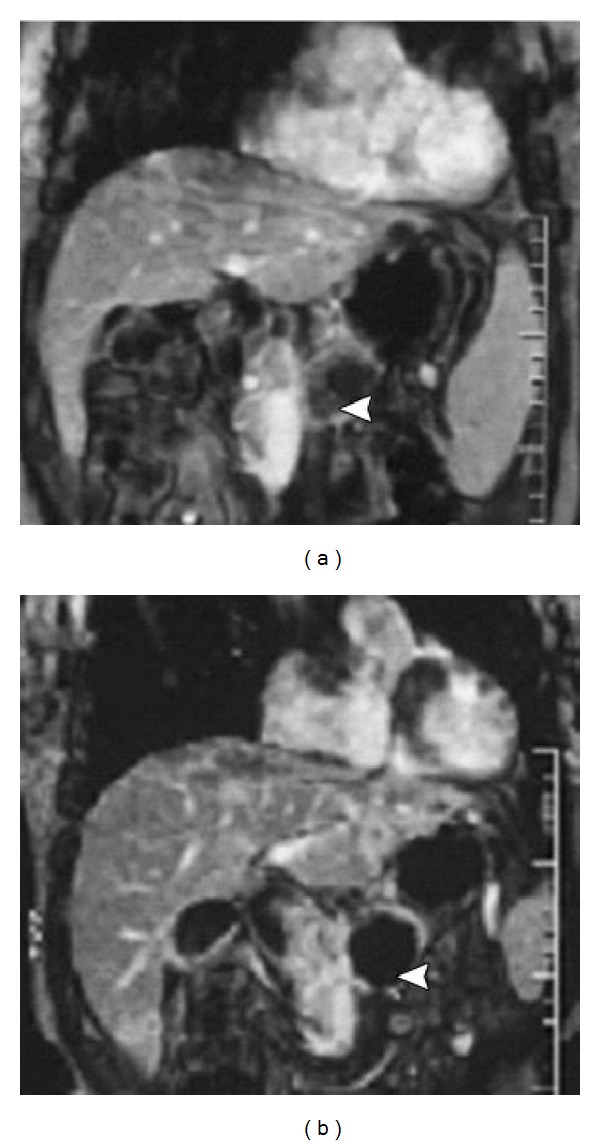
Dynamic contrast-enhanced gradient-echo T1-weighted MR images (a) before and (b) 2 weeks after HIFU ablation for advanced pancreatic cancer with a diameter of 4.5 cm. No evidence of contrast enhancement in the treated lesion (arrowhead) illustrates complete coagulation necrosis (used with permission [19]).

**Figure 7:**
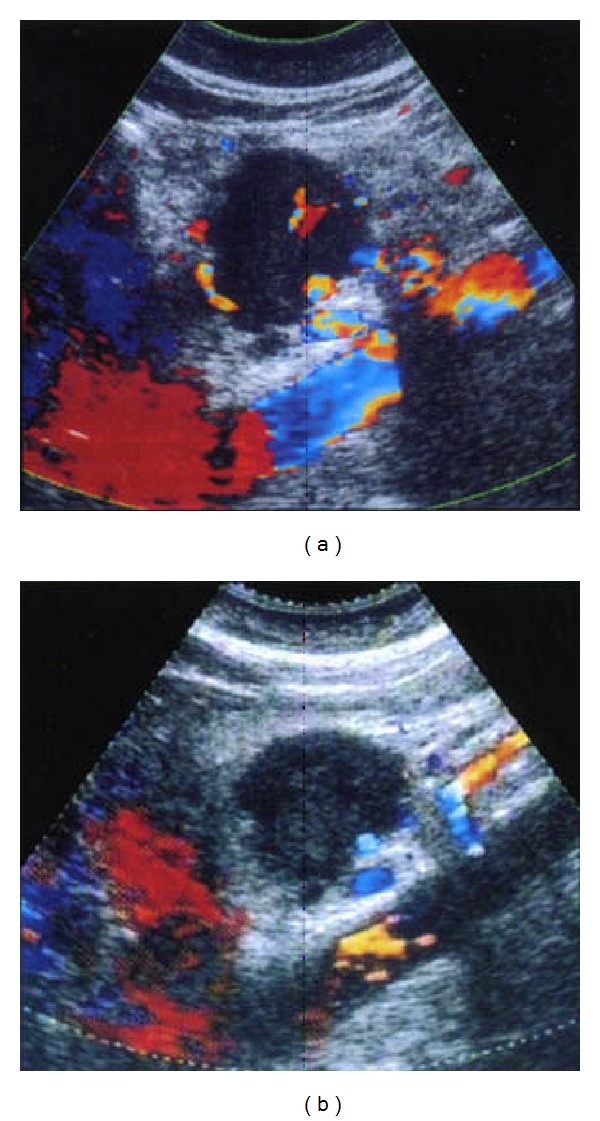
Sonography of pancreatic cancer (a) before and (b) after HIFU therapy showing the enhancement of echogenicity in the tumor but decrease of vascularity, an indicator of coagulative necrosis (used with permission [20]).

**Figure 8:**
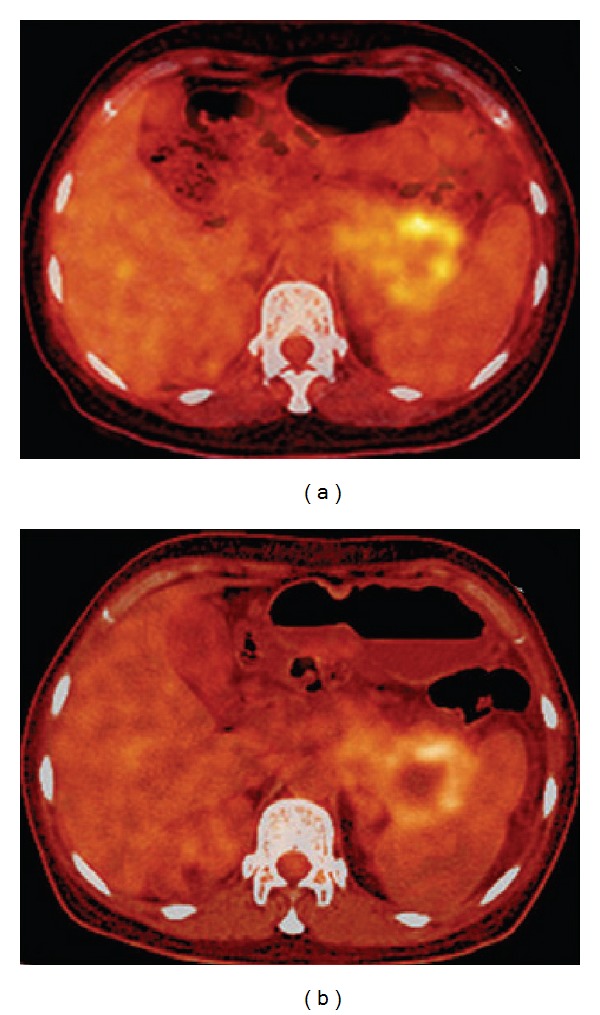
A PET-CT scan made (a) before HIFU demonstrates a SUVmax of  7.5 g/mL and (b) 3 months after HIFU demonstrates coagulative necrosis inside the tumor and the decreased SUVmax of 5.3 g/mL (used with permission [21]).

The purpose of posttreatment diagnosis is to verify the generation of necrosis in the target and its size. CT can clearly demonstrate the tumor size and shape as shown in Figure 5. But CT is insensitive to fat tissue, unreliable to assess the functionality of tumor's rim, and difficult for hypovascular tumor. Contrast-enhanced CT or multiple detector CT (MDCT) or magnetic resonance imaging (MRI) is also used before and after HIFU to assess necrosis by the absence of vascularity within the tumor, but not its metabolic activity. Iodinated contrast agents are prohibited to those who are allergic to iodine. Dose of contrast used in MRI is less than that of CT. Its slow injection rate may not cause discomfort or allergy to patients. Diagnostic capability becomes better with multiple dynamic scanning since MRI has nonionization as shown in Figure 6. T1-weighted MRI provides a good image contrast anatomy while T2-weighted one is sensitive to tumor coagulation and necrosis liquefaction. Ultrasound color Doppler is also sensitive to the blood flow inside the tumor as shown in Figure 7. Introduction of ultrasound contrast agent (e.g., microbubbles) could enhance the signal-to-noise ratio and diagnosis accuracy. The tumour size is not a reliable benchmark to evaluate HIFU efficacy in treating pancreatic cancer. Persistence of lack of enhancement suggests successful local tumor control. Contrast-enhanced MRI works excellently in the rapid assessment of therapeutic response of ablated tumor. PET or PET-CT is useful for diagnosing and staging of pancreatic cancer and for evaluating the outcome of HIFU treatment. Single-photon emission computed tomography (SPECT) as shown in Figure 8, a functional imaging, demonstrates the active metabolism of viable cancer cells. Real-time sonographic imaging is usually performed during the HIFU to examine the echogenicity in both the tumor and acoustic coupling path. However, hyperechoic changes in the target do not precisely correlate with the actual lesion.

**Figure 9:**
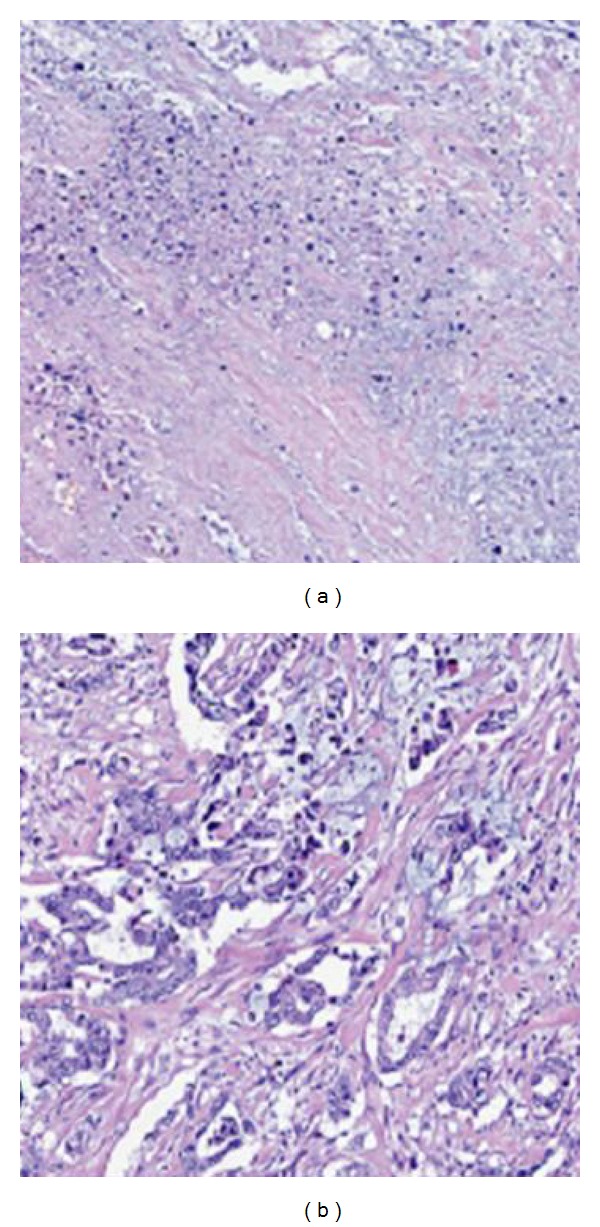
H&E staining of pancreatic cancer after HIFU ablation shows (a) disappearance of nuclei and necrosis and (b) the thermally fixed cancer cells (used with permission [22]).

In the peripheral region, the tumor cells are lethally damaged. In contrast, those thermally fixed in the central region look normal and similar to viable cells with the preservation of cellular structure as shown in Figure 9. Both electron microscopy and enzyme histochemical examination revealed an irreversible cell death. At autopsy, the tumor was replaced by a scar 10 months after HIFU ablation, and there was no apparent mass lesion remaining [18].

After clinical HIFU treatment, the serum amylase and urinary amylase levels are measured by a radioimmunometric assay as surrogate markers for traumatic pancreatitis. CA19-9, CA242, and CEA can be decreased by 49.41%, 34.93%, and 28.41%, respectively, which demonstrates the absence of pancreatitis as listed in Table 4.

## 4. Concurrent HIFU with Chemotherapy and Radiotherapy

Chemotherapy and chemoradiation (CRT) is an adjuvant treatment for resected pancreatic cancer but the primary one for locally advanced disease. Adjuvant chemotherapy with 5-fluorouracil or the combination of 5-fluorouracil, leucovorin, irinotecan, and oxaliplatin (FOLFIRINOX) can increase the 5-year survival (about 10 to 20%) in several large randomized studies. In contrast, adjuvant CRT is controversial with favoring practices in the USA but not recommended in Europe due to the absence of randomized studies. FOLFIRINOX is a new standard for advanced pancreatic cancer but has significant toxicities [29]. Safety data in patients with suboptimal status is not available, so caution should be taken in its use.

Advanced hypovascular tumors are more sensitive to heat shock due to no vascularity recovery after thermal injury. Thus, combination of HIFU with chemotherapy for advanced tumors is very attractive because the efficacy of chemotherapy is limited by long distance between tumor cells and blood vessels. After chemotherapy, but before HIFU ablation, an increase in blood flow is found inside the tumor using contrast-enhanced ultrasound (CE-US). Then, hypervascularity of the tumor is changed to hypovascularity by HIFU therapy, and the vasculature of large vessels through the tumor remains undamaged. As HIFU increases, the permeability of the vascular endothelial cells (maybe due to both intravascular cavitation and thermal effects) allows the chemotherapeutic agent to penetrate through the vessel into the interstitial space of the tumor, aids the distribution of the chemotherapeutic agent (pharmacokinetics) into the tumor due to the acoustic radiation force, and inhibits tumor cells to repair damage to chemotherapy [30]. Reduction of the vascularity through the tumor delays the drug clearance and increases the drug concentration. So ultrasound hyperthermia can reduce the dosage required and adverse effects of chemotherapy. Although the working principle of various chemotherapeutic agents and their targeted stage on cell metabolism and proliferation are different, combined HIFU and chemotherapy all result in a better outcome, high pain relief, CBR, and longer survival. In addition, intra-artery instead of vein injection can reduce the concentration in the circulating system and enhance the tolerability of patients.

HIFU ablation followed by radiotherapy is also a promising method. Reduced blood flow can prevent heat dissipation, lead to tumor cell damage and hypoxia, increase the cytotoxicity, and improve the sensitivity of radiotherapy. Radiotherapy is effective for oxygen-rich cells, and hyperthermia, in contrast, works well for hypoxic ones. Since fibrosis produced after hyperthermia influences radiation effects, HIFU is carried out after or simultaneously with the radiotherapy.

**Figure 10:**
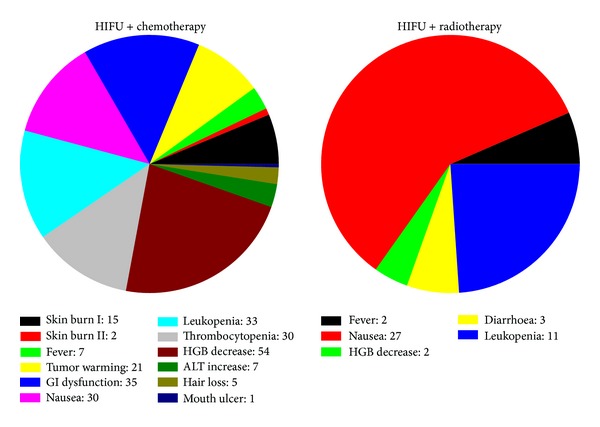
Summary of the complications found in the combination of HIFU ablation with chemotherapy and radiotherapy for advanced pancreatic cancer.

Clinical studies show that a combination of HIFU and chemotherapy or radiotherapy can achieve a higher CBR and longer survival than the single modality. The observed side effects are associated with HIFU, chemotherapy, and radiotherapy themselves. There is no enhancement in the complications by combining therapeutic modalities as shown in Figure 10. Chinese herbals were also used in conjunction with HIFU treatment [31, 32]. However, the number of cases is too small to fully evaluate its efficacy.

## 5. HIFU-Induced Immune Response

Recently, HIFU-induced immune response, suppression of the activity of tumor, and downregulation of tumor markers have attracted attention as an effective approach of cancer treatment. Selective recognition and destruction of tumor cells by the host immune system play an important role in antitumor immunity, which requires expression of tumor antigens. The immune system in most cancer patients fails to control the development and growth of initial cancer and to prevent local recurrence and metastasis after conventional therapies due to poor tumor antigen processing and immune-suppressive cytokines released by the tumor. HIFU can activate a host antitumor immunity to control micrometastasis and generate tumor resistance [33]. Increased NK cell activity, the population of CD4+ lymphocytes, and the ratio of CD4+/CD8+ in the blood circulation of cancer patients are found after HIFU ablation as listed in Table 5. Some clinical studies have shown greater concentrations of dendritic cells, macrophages, and B lymphocytes in the HIFU treatment group. Till now, the underlying mechanisms of antitumor immunity enhancement are not completely understood. Large amounts of tumor debris produced by HIFU can be released and reabsorbed in situ. A variety of tumor antigens remain in the tumor debris with and without typical characteristics of thermal damage. Circulating T cells activate specifically toward tumor antigens. High temperature unfolds the proteins from the native state to a more random state of lower organization, which can lead to either loss or preservation of antigenic determinants. Upregulation of heat shock protein (HSP) by hyperthermia can also stimulate the immune response. HIFU destructs the tumor structure and lowers its viability as well as the suppression of the immune system. Aseptic inflammation induced by pancreatic necrosis in HIFU ablation leads to the local accumulation of IL-1 and IL-2, which would adjust the antitumor immunity of host [34, 35].

## 6. Comments for Better Outcome

Although HIFU is an overall safe and noninvasive therapeutic modality for pancreatic cancer, it requires careful preoperative preparation as well as operative performance [36]. Understanding the factors for complications, recruiting appropriate patients, preparing the preoperation carefully, selecting proper HIFU operation parameters, and paying attention to adjacent vital organs during the procedure are necessary steps for minimizing severe complications [7]. Patients with extensive scars or scars lying in the path of the acoustic beam should be excluded because scar tissue absorbs ultrasound strongly and may result in a skin burn. Obstruction of bowel gas or bone to acoustic wave propagation towards the target should be removed to minimize the risk of causing unintended thermal injury. Therefore, the gas in the stomach and colon should be evacuated by careful bowel preparation, such as liquid food and no milk for 3 days, fasting for 12 hours before treatment, an enema in the early morning on the day of treatment, insertion of a urinary catheter (catharsis), and intraoperative bladders pressure. Drinking degassed water can remove the bowel gas quickly, but it has a short effective duration. Medicine may be more helpful, such as oral administration of quick-solution gastroenter-ultrasound developer. The skin at the wave entry site would be shaved to avoid the trapping of bubbles, degassed with a vacuum cup aspiration device, and degreased with 95% alcohol. Artificial pleural effusion may be placed if necessary to ensure the acoustic window. Proper positioning is selected by observing the acoustic path in the sonography. Applying slight abdominal pressure to the abdomen, such as using a soft water balloon, also helps to compress the bowel and clear the acoustic window. Respiratory motion during the treatment spreads the acoustic energy over a larger area in the target than expected and may result in incomplete tumor coagulation and damage to adjacent tissues. If it is too serious in operation, general anesthesia with endotracheal intubation and mechanical ventilation would be applied to allow provisional suspension of breath with controlled pulmonary inflation as well as reduction of pain and discomfort associated with HIFU ablation. Tracking the respiratory motion in real time would allow for rapid focus shifting in sync with the target position but needs proof in practice. Operators must monitor the imaging changes on adjacent vital organs, such as the myocardium, diaphragm, and bowel loops. Detection of the complications as early as possible allows the provision of appropriate and immediate management. A large-aperture transducer could decrease the acoustic intensity at the body surface and reduce the propensity of skin burn because of a wide convergent angle, which is a hypothesis that needs more clinical or* in vivo* evidence. If tumors are located in the pancreatic head, there remains a substantial possibility of biliary obstruction or biliary duct damage caused by the thermal ablation. An endobiliary stent should be routinely placed before HIFU ablation. At high power, each session should be within 1 hour. Lesions should cover the whole tumor area, and multiple sessions will be performed for satisfactory long-term outcome.

## 7. HIFU Challenges

Despite the large number of clinical cases of HIFU on advanced pancreatic cancer with promise, large-scale randomized and controlled trials at multiple centers with long-term follow-up have not been carried out to date to confirm these findings or to determine whether HIFU can improve overall survival by inducing local tumor response with or without chemotherapy, radiotherapy, or targeted drug [37]. Experiences in China may not be applicable to the Western countries. Appropriate HIFU treatment planning for complete coagulation but sufficient tissue margin is desirable in order to reduce the recurrence. Standard criteria are also required to evaluate both the short- and long-term efficiency and efficacy of HIFU on advanced pancreatic cancer. Pretreatment of HIFU on the margin of resectable pancreatic tumor may also be good for the better surgical outcome. A standardized dose of HIFU, chemotherapy, or radiotherapy has not been established, so current use is mostly empirical. An effective combination of treatment modalities is currently under investigation.

It seems clear that HIFU is finding its roles in clinics, although its technical development is still in its infancy [16]. Future developments will involve speeding up treatments and improving treatment targeting and monitoring. Motion artifact due to respiration and heartbeat is also a concern in clinics and needs to be monitored in real time for consistent delivery of HIFU energy during either end expiration or inspiration. An alternative solution is electrically steering the focus by the phased-array in order to keep the exposed target consistently. Ideally, the tissue inhomogeneity and attenuation can be compensated using phased-array design for accurate beam forming. In order to estimate the thermal dose, the acoustic output of the device, the acoustic and biological characteristics of the tumor, and the attenuation along the ultrasound pathway (primarily abdominal wall and viscera) are required [23]. One of the major factors that limit the wide application of HIFU is the absence of ultrasound-based thermometry and low frame rate and resolution of MRI-based one. HIFU system needs to be improved to work more appropriately for advanced pancreatic cancers.

## Figures and Tables

**Table 1:** Statistics of advanced pancreatic cancer patients treated with HIFU or in conjunction with chemotherapy or radiotherapy.

Men	Women	Age (year) (*n* = 3250)	Head	Body and tail	TNM-II	TNM-III	TNM-IV	Size (cm) (*n* = 339)
2014 (62.98%)	1184 (37.02%)	15–89 Mean: 60.8	1341 (53.68%)	1157 (46.32%)	69 (3.4%)	996 (49.14%)	962 (47.4%)	2–11.9 Mean: 4.76

**Table 2:** Statistics of the number of sessions, pain relief, clinical benefit rate, and survival of advanced pancreatic cancer patients undergoing HIFU therapy or in conjunction with chemotherapy or radiotherapy.

	Session	Pain relief	Complete relief (CR)	Partial relief (PR)	Clinical beneficial rate (CBR)	Survival (months)
HIFU	6.7 (*n* = 653)	71.33% (*n* = 1938)	29.66% (*n* = 1534)	39.83% (*n* = 1534)	71.06% (*n* = 508)	10.03 (*n* = 806)
HIFU + chemo	7.4 (*n* = 471)	59.72% (*n* = 602)	8.35% (*n* = 395)	45.39% (*n* = 395)	74.76% (*n* = 353)	10.16 (*n* = 270)
Chemotherapy		31.5% (*n* = 261)	4.31% (*n* = 100)	23.22% (*n* = 100)	38.85% (*n* = 222)	7.40 (*n* = 112)
HIFU + radio	5.2 (*n* = 130)	65.91% (*n* = 176)	27.84% (*n* = 176)	38.07% (*n* = 176)	82.15% (*n* = 89)	15.55 (*n* = 101)
Radiotherapy		29.65% (*n* = 67)	3.76% (*n* = 67)	25.89% (*n* = 67)	60.36% (*n* = 95)	

**Table 3:** Statistics of tumor size change, echogenicity in B-mode ultrasound image, and Karnofsky performance scale (KPS) score of advanced pancreatic cancer patients undergoing HIFU therapy.

Size decrease (*n* = 629)	Size increase (*n* = 629)	Echo (*n* = 186)	KPS % (*n* = 290)		
			Prior HIFU	Post HIFU	Increase
163 (25.91%)	57 (9.06%)	0%–100% Mean: 73.12%	38.1 ± 17.8~67.8 ± 9.4	74 ± 15~85.71 ± 4.95	151.77%

**Table 4:** Comparison of the serum levels before and after HIFU treatment.

	CA19-9 (U/mL)		CA242 (U/mL)		CEA (ng/mL)	
	Pre-HIFU	Post-HIFU	Pre-HIFU	Post-HIFU	Pre-HIFU	Post-HIFU
Range	42.6 ± 8.6~583.8 ± 20.4	21.5 ± 6.6~305.7 ± 19.3	73.6 ± 41.7~114.4 ± 42.0	46.3 ± 13.4~85.2 ± 21.9	38.4 ± 12.4~53.8 ± 17.3	18.9 ± 33~33.9 ± 14.8
Decrease	49.41% (*n* = 701)		34.93% (*n* = 135)		28.41% (*n* = 114)	

**Table 5:** Statistical summary of immune factors before and after HIFU ablation in pancreatic cancer patients.

	Pre-HIFU	Post-HIFU	Increase
CD3+ (*n* = 141)	37.39 ± 11.78~60.3 ± 5.9	51.8 ± 6.4~59.6 ± 6.7	112.94%
CD4+ (*n* = 93)	24.19 ± 7.02~32.6 ± 5.4	28 ± 10~34.7 ± 5.3	108.89%
CD4+/CD8+ (*n* = 93)	0.9 ± 0.3~1.1 ± 0.1	1.09 ± 0.53~1.4 ± 0.1	125.9%
NK (*n* = 28)	20.54 ± 9.1~21 ± 9	25 ± 13~25.52 ± 11.9	121.8%
